# In Situ TEM Study of Electrical Property and Mechanical Deformation in MoS_2_/Graphene Heterostructures

**DOI:** 10.3390/nano15020114

**Published:** 2025-01-14

**Authors:** Suresh Giri, Subash Sharma, Rakesh D. Mahyavanshi, Golap Kalita, Yong Yang, Masaki Tanemura

**Affiliations:** 1 Department of Physical Science and Engineering, Nagoya Institute of Technology, Gokiso-cho, Showa-ku, Nagoya 466-8555, Japan; cmx12001@ict.nitech.ac.jp (S.G.); rmahyavanshi@gmail.com (R.D.M.); 2 School of Chemistry, University of Bristol, Cantock’s Cl, Bristol BS8 1TS, UK; 3 Research and Development Laboratory, Nippon Denko Co., Ltd., Anan 774-0023, Japan; golapkalita@gmail.com; 4 State Key Laboratory of High-Performance Ceramics and Superfine Microstructures, Shanghai Institute of Ceramics, Chinese Academy of Sciences, 1295 Dingxi Road, Shanghai 200050, China; yangyong@mail.sic.ac.cn

**Keywords:** in situ TEM, chemical vapor deposition (CVD), folding, MoS_2_, mechanical manipulation

## Abstract

We present a versatile method for synthesizing high-quality molybdenum disulfide (MoS_2_) crystals on graphite foil edges via chemical vapor deposition (CVD). This results in MoS_2_/graphene heterostructures with precise epitaxial layers and no rotational misalignment, eliminating the need for transfer processes and reducing contamination. Utilizing in situ transmission electron microscopy (TEM) equipped with a nano-manipulator and tungsten probe, we mechanically induce the folding, wrinkling, and tearing of freestanding MoS_2_ crystals, enabling the real-time observation of structural changes at high temporal and spatial resolutions. By applying a bias voltage through the probe, we measure the electrical properties under mechanical stress, revealing near-ohmic behavior due to compatible work functions. This approach facilitates the real-time study of mechanical and electrical properties of MoS_2_ crystals and can be extended to other two-dimensional materials, thereby advancing applications in flexible and bendable electronics.

## 1. Introduction

The experimental discovery of graphene in 2004 [1] was a significant milestone that spurred extensive research into two-dimensional (2D) materials, including transition metal dichalcogenides (TMDCs). Due to their atomic-level thickness and large surface area, monolayer and few-layer TMDCs exhibit novel electronic, mechanical, and chemical properties that are not present in their bulk forms [2,3]. These materials are semiconductors with unique characteristics such as direct band gaps ranging from 1.37 to 1.96 eV, strong spin–orbit coupling, and high exciton binding energies [4]. These properties have been utilized in the fabrication of field-effect transistors, diodes, and solar cells [5,6,7], making them suitable for applications in electronics, optoelectronics, flexible devices, and energy-harvesting technologies [8].

Within the family of TMDCs, molybdenum disulfide (MoS_2_) has attracted significant interest due to its electron mobility, mechanical flexibility, and transparency. Atoms within a 2D layer are strongly bonded covalently, while van der Waals forces hold the layers together to form three-dimensional (3D) structures. The weak nature of van der Waals interactions allows the unique properties of individual atomic layers to be preserved in vertically stacked heterostructures, enabling the synthesis of structures with tailored electronic and material properties [9,10,11,12].

The development of chemical vapor deposition (CVD) techniques has accelerated research on 2D materials and heterostructures due to their scalability and controllability [13,14,15,16,17,18,19]. Inspired by the success of graphene, researchers are exploring new graphene-like 2D materials such as hexagonal boron nitride (h-BN), TMDCs, and MXenes for next-generation technological advancements [20,21,22,23]. Consequently, the folding of graphene and other TMDCs has been a topic of recent research interest in the field of electronics [24,25,26,27].

In pursuit of flexible and foldable electronics, numerous studies highlight bending- or-folding-driven devices that integrate mechanical deformability with high performance [28]. For instance, moisture-driven generators leverage folding to enhance ion transport for wearable power sources, while bioinspired foldable batteries retain performance over tens of thousands of strain cycles [29]. These findings underscore the importance of in situ bending/folding characterization for robust, wearable, and multifunctional electronics under demanding mechanical conditions.

Understanding the mechanical, chemical, and temperature-induced folding and wrinkle formation in 2D materials is essential for developing flexible and bendable electronics. Folding can significantly alter the physical and electronic properties of these materials by increasing bending stiffness and enabling them to adopt different shapes [30,31]. Various methods have been employed to induce folding in graphene, including growth on flat and curved substrates [32,33], mechanical force using atomic force microscope (AFM) tips [34,35,36], and surface functionalization followed by heat treatment [37,38].

AFM tips are convenient for manipulating or modifying graphene sheets by cutting and folding them in desired directions using controlled forces. However, AFM is limited in providing real-time information on nano-scale defects due to its limited scan size and slow scan speed. Transmission electron microscopy (TEM) has emerged as a versatile tool for the atomic-level characterization of 2D materials and their heterostructures [39,40,41]. TEM studies have revealed defects, doping, and bonding in 2D materials. By applying mechanical forces via specialized TEM holders, folding can be observed in real time at a high temporal resolution, enabling the study of dynamic processes and structural changes at the atomic level [42,43].

Conventional sample transfer processes for 2D materials and their heterostructures involve the etching of growth substrates followed by polymer-assisted transfer to TEM grids, which can cause unintentional doping and introduce impurities due to the multiple preparation steps involved [44,45]. Therefore, the transfer of pristine samples to TEM grids is highly desirable. Folding MoS_2_ crystals grown on the edges of graphite foils allows precise control over their alignment, which is crucial for studying their properties and advancing technologies [46].

While previous studies have explored in the situ folding of ultrathin MoS_2_, our work presents significant advancements by achieving transfer-free synthesis directly on graphite edges, ensuring the presence of pristine interfaces ideal for mechanical manipulation. Additionally, we integrate a TEM nanomanipulator with simultaneous electrical measurements, enabling the real-time observation of structural deformation and electrical behavior. Furthermore, our investigation of multiple folding–unfolding cycles provides new insights into defect formation and material resilience under repetitive mechanical stress, which are critical for applications in flexible and wearable electronics [33,45,47]. This integrated approach allows for the creation of folds, creases, and defects on MoS_2_ in situ, facilitating a thorough investigation of changes in electronic properties in real time without the need for multiple ex situ sample fabrication steps.

## 2. Materials and Methods

Growth of MoS_2_ Crystals:

Commercially available graphite foils, PERMA-FOIL®, TOYO TANSO Co. Ltd. (Nishiyodogawa-ku, Osaka, Japan) were cut into 2 mm × 5 mm pieces and used as substrates for the growth of MoS_2_ crystals via chemical vapor deposition (CVD). The edges of the graphite foils, composed mostly of few-layer graphene, provided a natural template for the orientation and alignment of the grown MoS_2_ crystals. Utilizing the edges avoids contamination and multiple transfer steps that are unavoidable for CVD-grown graphene on metallic substrates.

**Figure 1:**
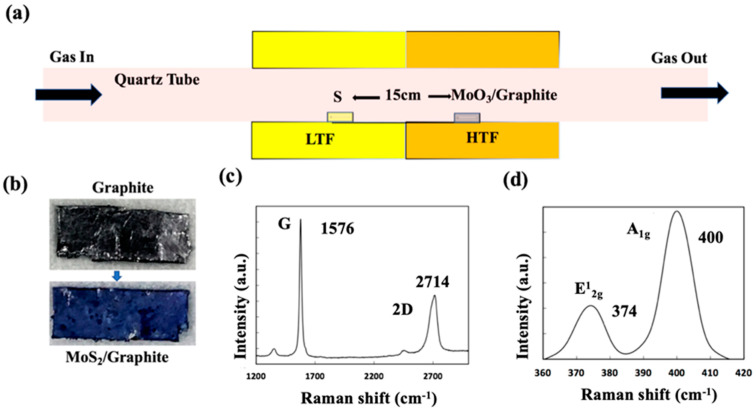
(**a**) Schematic of the CVD system used for MoS_2_/graphite synthesis. (**b**) Pictures of the graphite foil before and after MoS_2_ synthesis. The blue color observed after CVD indicates the deposition of MoS_2_. (**c**,**d**) Raman spectra measured on the MoS_2_/g sample, showing distinctive (**c**) G (1576 cm^−1^) and 2D (2714 cm^−1^) bands indicating graphite and (**d**) E^1^_2_g (374 cm^−1^) and A_1g_ (400 cm^−1^), which in turn indicate the presence of MoS_2_.

A dual-furnace CVD system was employed to independently control the temperatures necessary for MoS_2_ growth (Figure 1a). 1g Sulfur (S, 99.998% trace metals basis, Sigma-Aldrich, Product No. 213292, CAS No. 7704-34-9) powder was placed in the low-temperature furnace (LTF), maintained at 180 °C for sulfur evaporation. A mixture of argon and hydrogen gas (Ar/H_2_) at a ratio of 97:3 served as the carrier gas throughout the experiment. 100 mg Molybdenum (VI) oxide (MoO_3_, 99.97% trace metals basis, Sigma-Aldrich, Product No. 203815, CAS No. 1313-27-5) powder was placed in a ceramic boat in the high-temperature furnace (HTF), with a graphite foil positioned 2 cm upstream on the same boat. The distance between the sulfur and MoO_3_ sources was kept at 15 cm. The HTF was heated to 750 °C to facilitate MoO_3_ evaporation.

The evaporation timings of sulfur and MoO_3_ were carefully controlled to maintain appropriate vapor pressures, ensuring optimal MoS_2_ crystal growth. The growth time ranged from 10 to 30 min, depending on the desired coverage and size of the MoS_2_ crystals. After the growth process, the LTF was cooled first to condense any residual sulfur vapor, followed by normal cooling of the HTF to room temperature. The initially gray-colored graphite foil exhibited a bluish hue after MoS_2_ deposition (Figure 1b), indicative of successful growth.

Raman spectroscopy (NRS 3300 laser Raman spectrometer with a laser excitation energy of 532.08 nm) was performed to characterize the as-synthesized MoS_2_/graphite samples. Peaks corresponding to graphite (G peak at 1576 cm^−1^ and 2D peak at 2714 cm^−1^) and MoS_2_ (E^1^_2g_ and A_1g_ peaks at 374 cm^−1^ and 400 cm^−1^, respectively) were observed (Figure 1c,d), confirming the presence of the MoS_2_/graphite heterostructure [48]. Raman characterization was conducted on thicker parts of the sample, as atomically thin MoS_2_/graphene at edges is not easily distinguishable using Raman spectroscopy due to the large spot size.

All in situ experiments were performed on a JEOL JEM ARM-200F (JEOL Ltd., Tokyo, Japan) transmission electron microscope operated at 200 kV. The Supplementary Videos S1 and S2 were captured using a Gatan UltraScan CCD camera, (Gatan, Inc., Pleasanton, CA, USA) recording at about 5–10 frames per second with LiteCam software (Version 3.9). A small portion of the MoS_2_/graphite sample was carefully cut and directly mounted onto a TEM holder without any additional treatment or transfer steps. TEM imaging was performed on the edges of the graphite foil, which consisted of few-layer graphene, allowing the electron beam to transmit through the sample and enabling an observation of the morphology, structure, and distribution of the MoS_2_ crystals.

A nano-manipulator equipped with a tungsten (W) probe, controlled via a piezo-based microcontroller, was used to apply mechanical force to the MoS_2_ crystals within the TEM. This setup also allowed for the measurement of electrical properties of the MoS_2_/graphite heterostructure by applying a bias voltage through the W probe [49,50]. This integrated system enabled the real-time observation of folding, wrinkling, and tearing processes at high temporal and spatial resolutions.

## 3. Results and Discussion

**Figure 2:**
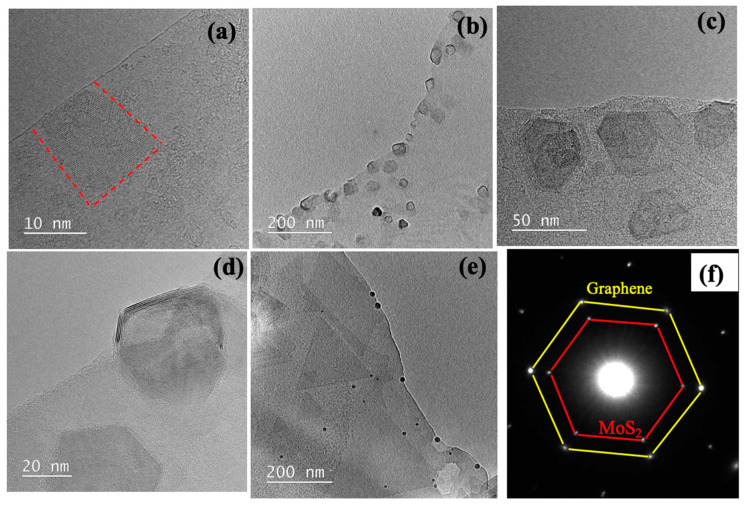
(**a**,**b**) TEM images showing MoS_2_ crystals on a graphene edge. Red dotted line in Figure (**a**) shows very thin MoS_2_ crystal on Graphene edge (**c**,**d**) TEM images showing hexagonal MoS_2_ on graphene. (**d**) MoS_2_ crystal partially on graphene and partially suspended, exposing individual layers. (**e**) Triangular MoS_2_ flake on graphite. (**f**) Selected area diffraction pattern of a typical MoS_2_/graphene heterostructure.

Low-magnification TEM images revealed that MoS_2_ crystals were dispersed on few-layer graphene substrates, with crystals growing along the edges, as indicated by dotted lines in Figure 2a. The crystals were predominantly hexagonal (Figure 2b,c), although some triangular-shaped MoS_2_ crystals were also observed (Figure 2e). The triangular MoS_2_ crystals exhibited identical alignment on the graphene substrate, indicating epitaxial growth. According to the published literature, the morphology of MoS_2_ crystals can be tuned by adjusting the growth temperature and the position of the growth substrate [51]; however, this optimization is beyond the scope of the current work.

Figure 2d shows that MoS_2_ crystals initially nucleate on graphene but extend beyond the substrate, forming a freestanding structure. This behavior suggests that MoS_2_ growth follows an edge epitaxy mechanism rather than being strictly substrate-dependent, consistent with observations in similar systems [46,52]. Under our CVD conditions, the under-coordinated sites or step edges on graphite (i.e., zigzag or armchair edges) act as favorable nucleation points for MoS_2_. After nucleation, the MoS_2_ domains expand outward, often resulting in partially freestanding crystals extending beyond the substrate. A similar mechanism has been reported by Lu et al. [53], who found that MoS_2_ nucleates and grows from graphite step edges, with the edge structure determining the island orientation. This edge-driven process explains both the coherent lattice alignment of MoS_2_ on graphite and the subsequent overgrowth beyond the substrate boundaries. The selected area electron diffraction (SAED) pattern presented in Figure 2f reveals two sets of hexagonal diffraction spots: the outer hexagons (yellow hexagon) correspond to graphene, while the inner hexagons (red hexagon) represent MoS_2_. The perfect alignment of these hexagonal patterns confirms that the MoS_2_ crystals grow on graphene without rotational misalignment, demonstrating precise epitaxial growth. This epitaxial relationship is crucial for applications requiring coherent interfaces between different 2D materials [33].

**Figure 3:**
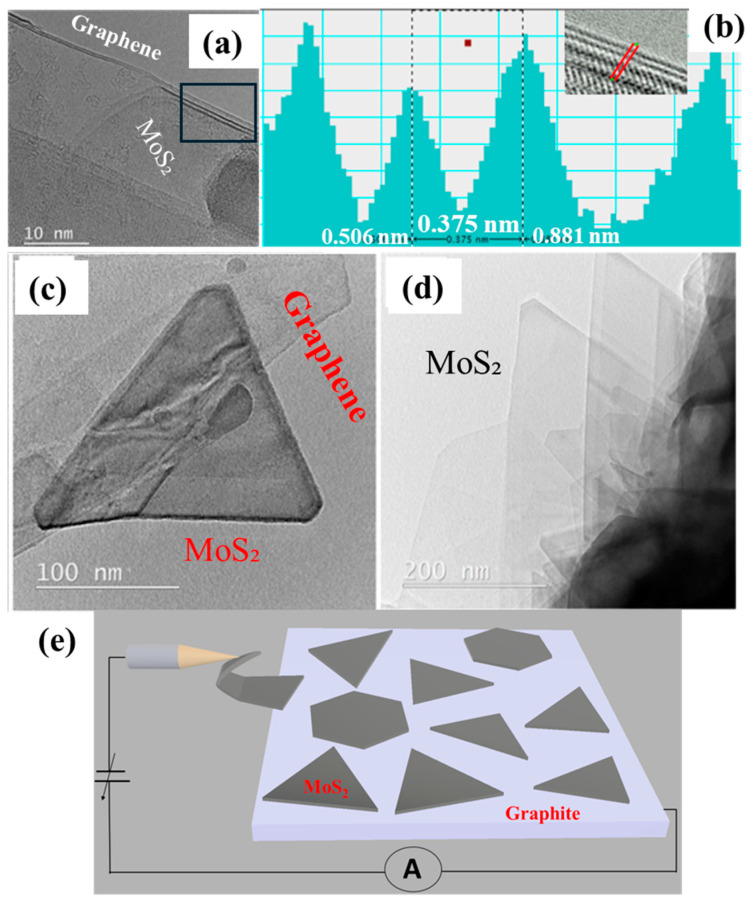
(**a**) TEM image showing bilayer MoS_2_ on bilayer graphene. (**b**) Line profile measured across the MoS₂/graphene layers in the rectangular region marked in (**a**). The inset shows the interlayer distance between graphene and MoS₂, measured as 0.375 nm. (**c**) Triangular MoS_2_ crystal growing on graphite with a suspended edge. (**d**) Array of freestanding triangular MoS_2_ crystals on graphene. (**e**) Schematic diagram showing the in situ TEM setup for the electrical measurement of the MoS_2_/graphene heterostructure.

In this study, we focused on the direct growth of MoS_2_ on graphite edges to preserve its pristine nature for in situ TEM investigations. While this method avoids contamination or defects introduced during conventional transfer processes, it presents challenges for practical applications where the MoS_2_ must be transferred to other device platforms. The strong van der Waals interactions between MoS_2_ and graphite, along with the difficulty in chemically or mechanically separating these materials without damaging the epitaxially grown layers, complicate transfer attempts. Figure 3a shows the edge of a bilayer graphene sheet with MoS_2_ grown on it, forming a vertical heterostructure. Line profile measurements across the edge indicated an interlayer distance of approximately 0.375 nm (Figure 3b), an intermediate spacing between the interlayer spacings of graphene (~0.335 nm) and MoS_2_ (~0.615 nm), suggesting an interface with unique structural characteristics. This intermediate spacing may result from van der Waals interactions at the heterojunction [54]. Larger MoS_2_ crystals are preferable for electrical property measurements and mechanical manipulation due to their easier handling. Figure 3c shows a freestanding triangular MoS_2_ crystal growing on the graphene edge, indicating that the growth mechanism does not change even when there is no underlying substrate. Figure 3d shows an array of triangular MoS_2_ crystals growing on graphite. For electrical property measurements and manipulation using the probe, larger and freestanding MoS_2_ crystals are preferred. Figure 3e illustrates the schematic design of the in situ TEM setup, showing the position of the probe and the location of the freestanding MoS_2_ crystal.

**Figure 4:**
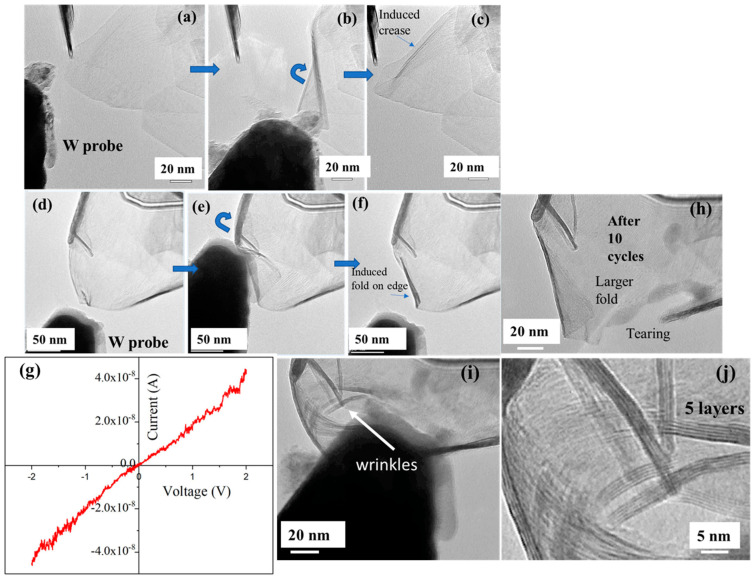
(**a**–**c**) Video clips from Video S1 showing the folding process of MoS_2_ with the formation of a crease. (**d**–**f**) Video clips from Video S2 showing the folding process of MoS_2_ with the formation of a fold. (**g**) I–V characteristics of the MoS_2_/graphene heterostructure showing near-ohmic behavior. (**h**) Formation of a larger fold and tearing of MoS_2_ after 10 cycles of folding. (**i**) Pushing MoS_2_ with a W probe to demonstrate wrinkle formation (in the region pointed by the arrow), with individual layers clearly visible in the high-magnification image (**j**).

After selecting a suitable MoS_2_ crystal, a nanomanipulator was used to position the W probe near the crystal, as shown in Figure 4a. The probe was manipulated to apply mechanical force to the crystal [55,56]. The interaction of the probe and the induced folding were recorded and are shown in Supplementary Videos. Figure 4a–c show screenshots taken from Supplementary Video S1, depicting the approach of the probe, the formation of a fold, and the retraction of the probe. The approaching probe makes contact with the edge of the freestanding crystal and is gradually pushed upward, causing the formation of wrinkles. Upon retracting the probe, the MoS_2_ is left for observation. Comparing Figure 4a,c, it is evident that the MoS_2_ retains its original morphology without significant visible deformation. However, the initial linear, faintly visible wrinkle becomes more pronounced and darker, as shown in Figure 4c, indicating the mechanical stress-induced enhancement of the wrinkle.

We repeated an identical experiment with another MoS_2_ crystal, as shown in Supplementary Video S2. Screenshots from the video are presented in Figure 4d–f. In contrast to the earlier experiment, mechanical stress-induced folding of the MoS_2_ edge is clearly visible in this case. The formation of wrinkles or folds may depend on factors such as the direction of the applied force and the thickness of the crystal [57,58]. After the formation of a minor fold on the crystal edge (Figure 4f), additional force was applied with the probe, followed by retraction. After 10 cycles, significant deformation and changes in the morphology of the crystal were observed, as shown in Figure 4h. A large fold is apparent on the crystal edge, along with clear tearing of the crystal at the lower part. These observations suggest that repeated cycles of stress introduce defects in the crystals, leading to the formation of permanent larger folds and tears, consistent with the loss of elasticity under prolonged mechanical deformation [47].

During the application of mechanical force, wrinkle formation was observed at multiple locations, as shown in Figure 4i. Figure 4j. shows a highly magnified TEM image of the area indicated by the arrow in Figure 4i. Individual MoS_2_ layers are clearly visible, with the number of layers ranging from 5 to 10. In TEM observations of 2D materials, wrinkles and folded edges are ideal sites for measuring the number of layers due to the contrast variation and electron diffraction effects [59]. By using probe-induced mechanical force, wrinkles can be artificially induced, and the thickness can be measured, providing a method for the in-situ characterization of layer numbers. While this study primarily focuses on demonstrating the in situ folding and electrical characterization of MoS_2_/graphene heterostructures, we acknowledge that the number of MoS_2_ layers can significantly influence folding performance, including bending radius and susceptibility to fracture. Investigating thickness-dependent mechanical behavior presents an important avenue for future research to further understand and optimize the resilience of MoS_2_-based flexible electronics [58,59].

In addition to mechanical manipulation, a bias voltage was supplied through the W probe to measure the electrical properties of the MoS_2_ crystal. Figure 4g shows the I–V characteristics of the MoS_2_ crystal when a bias voltage ranging from −2 V to +2 V was applied. The I–V curve exhibits a near-ohmic characteristic, indicating low-resistance contact between the probe and the MoS_2_ crystal. The W probe, with a high work function of approximately 4.5 eV, closely matches the electron affinity (χ = 4.22 eV) of multilayer MoS_2_, leading to the formation of near-ohmic behavior [60]. This method can also be useful for studying Joule heating-induced electrical and morphological transformations of MoS_2_ in situ by supplying higher bias voltages, which can induce phase transitions or defect formation [61]. While the I–V curve in our study shows near-ohmic behavior, some studies, such as Jahangir et al. [62], have reported Schottky behavior in transfer-free MoS_2_/CVD graphene interfaces. The differences in contact behavior may arise from variations in layer thickness, doping levels, and substrate conditions. In our case, the nearly ohmic response is likely due to the clean, defect-free interface achieved through the direct epitaxial growth of MoS_2_ on graphite edges, which eliminates the need for transfer processes that could introduce contaminants or defects.

Our results demonstrate that the mechanical manipulation of MoS_2_ crystals using an in situ TEM setup allows for the real-time observation of structural changes, such as folding, wrinkling, and tearing, under controlled mechanical stress. The ability to induce and study such deformations at the nanoscale is crucial for understanding the mechanical properties of 2D materials and their potential applications in flexible electronics [63]. Additionally, the simultaneous electrical measurements provide insights into how mechanical deformations affect the electrical properties, which is important for device applications where mechanical flexibility is required [64]. This approach offers a powerful platform for investigating the interplay between mechanical and electrical properties in 2D materials, paving the way for the development of next-generation flexible and wearable electronic devices.

## 4. Conclusions

We developed a straightforward and effective method for synthesizing MoS_2_ crystals on graphite foil edges, resulting in high-quality MoS_2_/graphene heterostructures with precise epitaxial alignment. The freestanding MoS_2_ crystals can be mechanically manipulated and electrically characterized in real time using an in-situ TEM setup equipped with a nano-manipulator and W probe. This integrated approach enables the study of folding, wrinkling, and tearing processes, as well as the measurement of electrical properties. Our method holds significant potential for advancing the understanding and application of 2D materials in flexible and bendable electronics, and can be extended to other materials and device architectures.

## Data Availability

Data are contained within the article and Supplementary Materials.

## Supplementary Materials

The following supporting information can be downloaded at https://www.mdpi.com/article/10.3390/nano15020114/s1: Videos S1 and S2: In situ TEM videos.
